# Bioluminescent detection of isothermal DNA amplification in microfluidic generated droplets and artificial cells

**DOI:** 10.1038/s41598-020-78996-7

**Published:** 2020-12-14

**Authors:** Patrick Hardinge, Divesh K. Baxani, Thomas McCloy, James A. H. Murray, Oliver K. Castell

**Affiliations:** 1 Cardiff School of Biosciences, Cardiff University, Sir Martin Evans Building, Museum Avenue, Cardiff, CF10 3AX UK; 2 Cardiff School of Pharmacy and Pharmaceutical Sciences, Cardiff University, Redwood Building, King Edward VII Avenue, Cardiff, CF10 3NB UK; 3 Cardiff School of Engineering, Queen’s Buildings, 12-17 The Parade, Cardiff, CF24 3AA UK; 4 Molecular Sciences Research Hub, Imperial College London, 80 Wood Lane, Shepherd’s Bush, London, W12 0BZ UK

**Keywords:** DNA, Biomedical engineering, Biophysical chemistry, Biochemistry, Biotechnology, Chemical biology, Molecular biology, Engineering, Biological techniques, Analytical biochemistry, Imaging, Lab-on-a-chip

## Abstract

Microfluidic droplet generation affords precise, low volume, high throughput opportunities for molecular diagnostics. Isothermal DNA amplification with bioluminescent detection is a fast, low-cost, highly specific molecular diagnostic technique that is triggerable by temperature. Combining loop-mediated isothermal nucleic acid amplification (LAMP) and bioluminescent assay in real time (BART), with droplet microfluidics, should enable high-throughput, low copy, sequence-specific DNA detection by simple light emission. Stable, uniform LAMP–BART droplets are generated with low cost equipment. The composition and scale of these droplets are controllable and the bioluminescent output during DNA amplification can be imaged and quantified. Furthermore these droplets are readily incorporated into encapsulated droplet interface bilayers (eDIBs), or artificial cells, and the bioluminescence tracked in real time for accurate quantification off chip. Microfluidic LAMP–BART droplets with high stability and uniformity of scale coupled with high throughput and low cost generation are suited to digital DNA quantification at low template concentrations and volumes, where multiple measurement partitions are required. The triggerable reaction in the core of eDIBs can be used to study the interrelationship of the droplets with the environment and also used for more complex chemical processing via a self-contained network of droplets, paving the way for smart soft-matter diagnostics.

## Introduction

The ability to amplify, detect with high sensitivity and quantify specific nucleic acid sequences is an important and well established sector of the multibillion dollar molecular diagnostic industry which is expected to continue to grow to a market of 12.5 billion US dollars^1^ by 2025. The benchmark DNA amplification method is the quantitative polymerase chain reaction (qPCR) which detects and quantifies DNA target sequences in real time. The ability to increase the quantity of specific DNA sequences has enabled the detection of particular traits that would otherwise remain undetected and has benefitted a wide range of areas from infectious disease diagnosis and oncology, to the identification of genetically modified organisms, and “DNA fingerprinting” in forensic science. In low resource settings and at point-of-care this is particularly desirable. To do this a thermostable polymerase enzyme is required to synthesize nascent DNA through cycles of temperature change. The Covid-19 pandemic has also highlighted the challenges with mass, centralised, qPCR diagnostics in otherwise well-resourced nations, where readily scalable point-of-care diagnostics could provide an alternative^2^.

**Figure 1:**
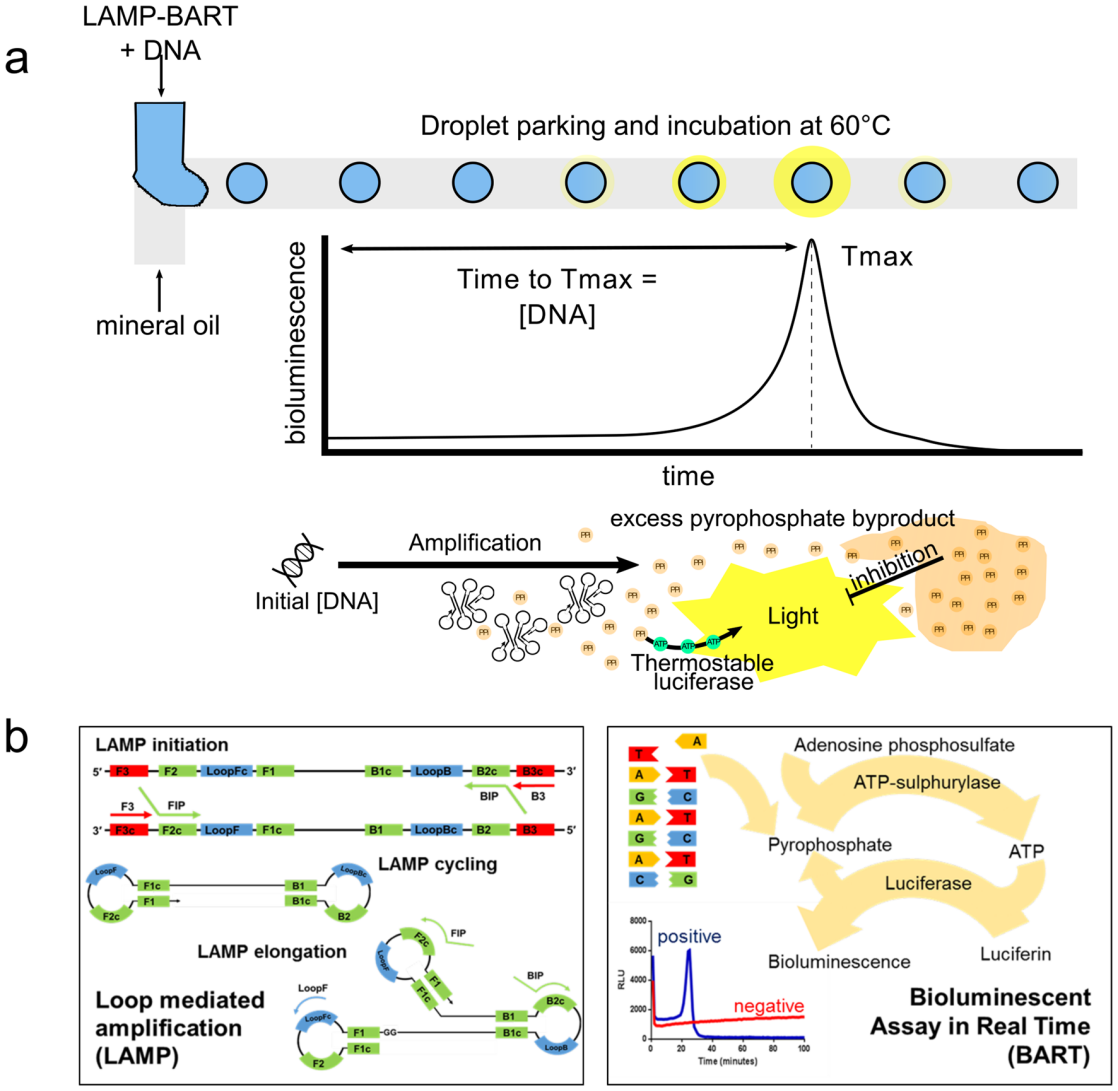
Schematic of LAMP BART reaction and droplet generation. (**a**) Schematic of LAMP BART bioluminescent droplet generation; aqueous LAMP BART droplets are formed at a microfluidic T-junction with an immiscible oil phase. Upon heating, the increase in bioluminescence from target DNA containing droplets is characterized by a peak in light output before inhibition by an excess of the by-product of DNA amplification, pyrophosphate, (**b**) simplified loop-mediated amplification (LAMP) schematic showing the three main stages, amplification initiation, cycling of “dumbbell” structures and elongation of looped concatemers, representation of the bioluminescent assay in real time (BART) showing the conversion of pyrophosphate into light and BART profiles for positive and negative samples.

Due to the challenges posed by the requirement of thermocycling, notably challenging in a microfluidic context, there has been increasing interest in the development of isothermal DNA amplification methods^3–5^. The most widely reported method is loop-mediated amplification (LAMP)^6^ which has been shown to be rapid, highly specific^7^ and sensitive^8^, with a greater tolerance to PCR inhibitors^9–11^ frequently encountered in clinical samples, thus allowing less stringent DNA extractions or sample clean up. In principle throughput of this method can be increased using droplet microfluidics. A schematic of LAMP amplification is shown in Fig. 1 together with the concept of its application in droplets. In principle detection of as little as a single copy of DNA is achieved via loop-amplification followed by detection of subsequently emitted light. Briefly, loop forming primers FIP and BIP (forward and backward inner primers) invade the double stranded DNA and are extended by a displacement polymerase enzyme. The newly formed DNA is displaced by further primers B3 and F3 to initiate amplification, with continuation of this process leading to the formation of complex looped structures. The LAMP reaction can also be accelerated with additional Loop^12^ or STEM^13^ primers. A by-product of DNA synthesis is inorganic pyrophosphate (PPi) produced in significant exponential quantities by the prolific LAMP reaction, and which can subsequently be used for detection.

The high concentration of pyrophosphate generated by LAMP is ideally suited towards bioluminescent assay in real time (BART) reporter^14^ quantification. Here, the pyrophosphate is catalysed into adenosine triphosphate (ATP) in an ELIDA reaction^15^ which is then converted to bioluminescence through a thermostable luciferase reaction (Fig. 1a). Quantification of DNA is realized here via the production of a peak in light intensity, of which timing is inversely proportional to the original DNA template concentration^14^. Upon realization of the bioluminescent peak, inhibition of bioluminescence occurs rapidly as a combined result of the depletion of adenosine-5′-O-phosphosulfate (APS) which is required to convert the PPi to ATP and the concomitant accumulation of PPi which also inhibits luciferase at high concentrations. Light output from a positive sample is thereby characterised by a growth in the rate of light emission as DNA is amplified before falling below the baseline of negative controls due to luciferase inhibition or APS depletion. Negative samples are characterised by a continuous baseline level of bioluminescence. BART is not appropriate for end-point detection due to the observation that the light output from the positive sample can be higher or lower than the negative sample at any one time point.

In contrast with PCR, LAMP–BART reactions can typically be performed isothermally at 60 °C, and can produce real-time results within a few minutes that are easily detected by photodiode or simple CCD camera imaging. Furthermore, BART detection is closed tube format which is highly applicable for use in an encapsulated droplet chemistry, whilst also reducing the risk of amplified DNA contamination. Also LAMP–BART has been employed to detect DNA template at the single molecule level^8^ and has recently been shown to be applicable to single and low copy number quantification^16^. These features provide significant benefits over the more commonly used PCR in terms of ease of quantification and sample amenability, with no compromise in measurement sensitivity for point-of-care application.

Microfluidic approaches have long been applied to DNA amplification methods, most notably PCR amplification. Beer et al.^17^ demonstrated the ability of single copies of DNA to be PCR amplified and detected in 10 picolitre droplets. In this way, absolute quantification of initial DNA template is achievable by using multiple droplets at low copy number and applying Poisson statistics^18^. In such digital PCR (dPCR), each droplet originating from a sample can be considered its own micro-assay, and thus mass throughput can be achieved by rapidly producing microfluidic droplets creating multiple compartments of a single sample^19^. In addition multiplexing probes via the use of different fluorophores, or different fluorescent intensities per probe can expand assay capacity^20^. Due to the benefits offered by dPCR vs qPCR^21^, the technology is now commercialised and has occupied the niche of ultra-sensitive DNA template quantification^22^.

In microfluidic PCR, thermocycling remains an important challenge which at times can be exacerbated by miniaturization and microfluidic implementation^23^, alongside challenges in device integration^23,24^. This is particularly challenging in dPCR where homogenous amplification is essential. For these reasons, high-sensitivity, isothermal methods of DNA amplification such as LAMP–BART provide an alternative, with potential to simplify operation and implementation, as well as affording opportunity to operate without sample clean-up. To date proof-of-concept demonstrations of isothermal DNA amplification via LAMP in single phase microfluidic systems have been demonstrated^25–28^, as well as demonstrations of droplet-based LAMP^29–31^, and a number of paper-based microfluidic LAMP devices^32–35^, including more recently for Covid-19^36^. However, to date, this has been in the absence of an integrated real-time quantitative readout e.g. in the form of a coupled biochemical or bioluminescent reaction such as BART. In this context, only two examples of microfluidic LAMP–BART exist to date^37,38^, both describing on-chip single phase approaches with a single sample. As such, there is significant opportunity for the scaling of LAMP–BART approaches using droplet microfluidics to create multiple segregated low-volume assay containers for high-throughput, quantitative assessment.

There is much emerging interest in harnessing the advantages afforded by microfluidic compartmentalisation for biochemical reactions within more sophisticated multicompartmentalised, or biochemically fuctionalised artifical cells^39–41^. With anticipation of such systems, inspired by biological cells, forming a next generation of smart soft-matter devices, with applications spanning the development of smart therapeutic delivery systems and freestanding smart biosensors^42^. The ability to identify, amplify and respond to target nucleic acid sequences in such systems opens opportunities for both innovative diagnostics and would provide new chemical processes that can link to other biochemical mechanisms in such systems, increasing the scope of functionality.

Multicompartment droplet architectures incorporating lipid bilayers akin to biological membranes^40,43^ represent a promising chassis system allowing harnessing of lipid bilayer barrier properties and incorporation of functional membrane proteins. DNA expression through in vitro transcription and translation (IVTT) has been demonstrated in such droplet interface bilayers systems, along with the ability to incorporate subsequently expressed protein channels in their lipid bilayers^44–46^, enabling selective communication between compartments. Such membrane segregated aqueous droplet networks may be encased within a hydrogel shell^47^, allowing them to be stable and freestanding in an aqueous environment, and stored for later use. The presence of lipid bilayers between the internal droplets and the external environment establishes a route for the controlled biomimetic communication between internal and external environments^48^. As such, there is a significant opportunity for the incorporation of LAMP–BART DNA amplification and detection into artificial cells of this nature. This may enable freestanding micro DNA quantification vessels for sample analysis outside of laboratory environments, and also serve as a synthetic biology tool to enable DNA amplification and biochemical process coupling, in this case light emission, in response to small quantities of target biomolecules.

Here, the amplification and bioluminescent detection of DNA template using LAMP–BART in microfluidically generated droplets is demonstrated. A gradient of DNA concentrations across a series of droplets measured in parallel demonstrates the light emission dependency on DNA concentration, providing the opportunity for quantitative, low volume, high-throughput assays. Furthermore, the ability to perform LAMP–BART within the internal aqueous droplets of eDIB artificial cells is demonstrated quantifying light emission from individual cores, paving the way for free-standing soft-matter analytical devices.

## Results

### LAMP–BART droplets in tubing

**Figure 2:**
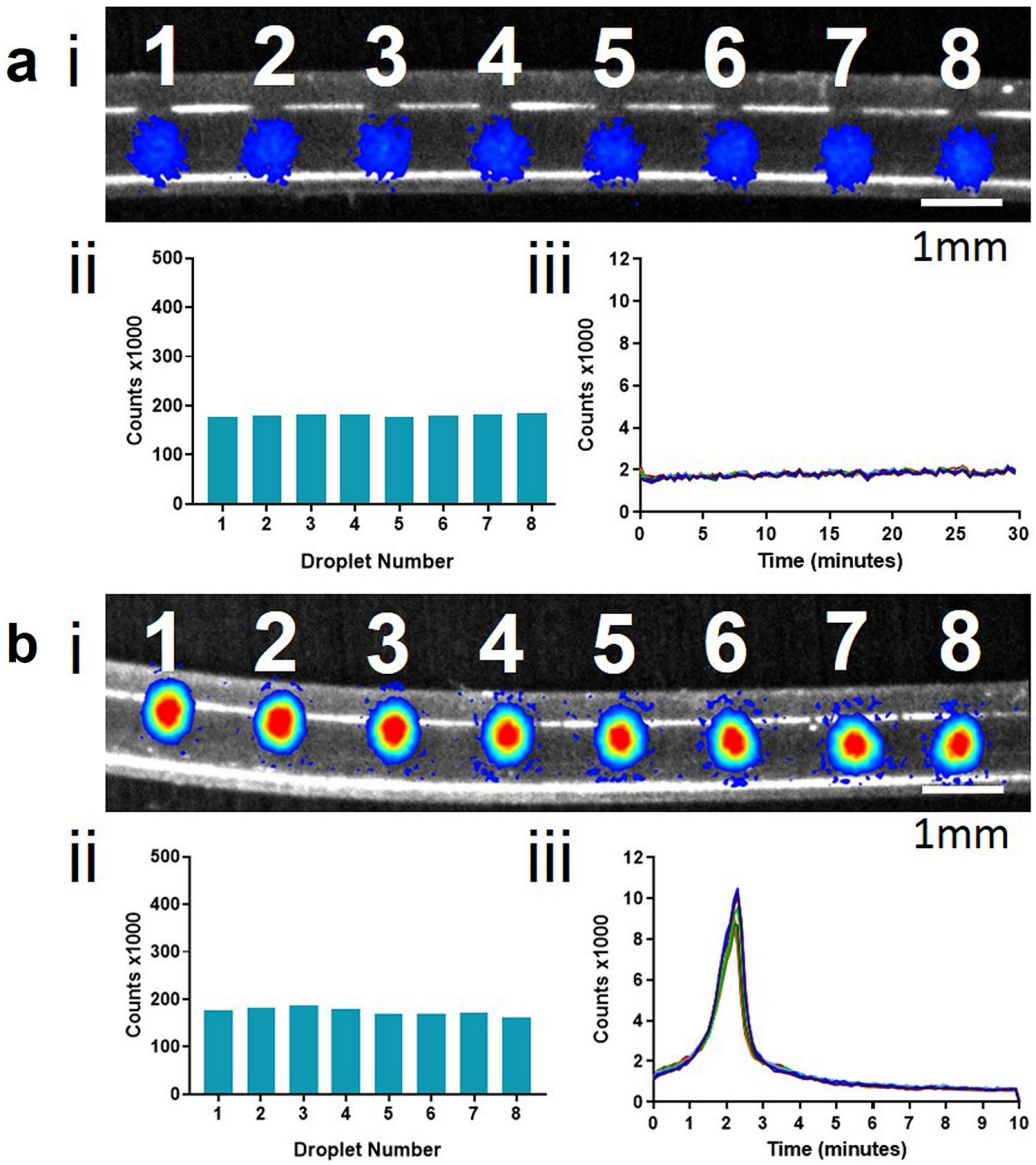
LAMP–BART droplets in the absence (**a**) and presence (**b**) of DNA template. Artificial template (NOSt) at 1 × 10^8^ copies per droplet with NOSt LAMP primers. (**a**) LAMP–BART no template control (NTC) in droplets, (**b**) LAMP–BART with DNA template. (i) Pre-acquisition image showing the position of droplets within the microfluidic tubing overlaid with maximum photon counts from the bioluminescent reaction within the droplets. A high intensity maximum is observed in the presence of DNA template (**b**) (ii) total photon counts for each droplet number indicate comparable total photon yields indicative of the uniformity of droplet size and homogeneity of low volume reactants. (iii) Real time photon counts from individual droplets show a rapid peak in light intensity with the inclusion of DNA template (**b**), which is absent in droplets without DNA (a) (Supplementary Information, Videos 1 and 2). The target droplet diameter was 500 micrometers. Variation in droplet size is shown in Supplementary information, Sect. 3. The limit of quantification of the NOSt artificial DNA template with the LAMP–BART assay was calculated to be approximately 4 copies per droplet (SI Figs. S1 and S2).

LAMP–BART droplets without DNA template or with template were parked in tubing for analysis. Two LAMP assays were used targeting DNA sequences for the 35S promoter (35Sp) and NOS terminator (NOSt), both of which have been extensively studied for sensitivity and specificity in previous publications^7,8,11,16^. Microfluidic droplets of LAMP–BART reagents with or without DNA template at 1 × 10^8^ copies of the NOSt DNA template per droplet were generated and parked within tubing (Fig. 2). The flow rates of 4.0 millilitres per hour for the LAMP–BART reagents and 9.0 millilitres per hour for the mineral oil ensured a steady production of evenly spaced droplets. The DNA amplification reaction was heat activated and maintained and imaged at 60 °C. The limit of detection (LOD) for LAMP–BART droplets incorporating NOSt artificial DNA template is shown in Supplementary Information Figs. S1 and S2.

The total photon counts for each set of eight droplets averaged 1.81 × 10^5^ counts ± 0.03 × 10^5^ for samples without DNA and 1.75 × 10^5^ counts ± 0.08 × 10^5^ for samples with DNA. Samples both with and without DNA template gave rise to background levels of bioluminescence below the 2000 count level. Samples containing DNA template rapidly increased in bioluminescence giving rise to a peak light intensity (average Tmax 2.25 ± 0.05 min), from which light output rapidly reduced to below background levels. All eight droplets gave rise to a bioluminescent peak within 3 min of heating, with little variation in peak light intensity. LAMP amplification of the DNA template and the BART detection were both achieved from the nanoliter volume of the droplets and no LAMP amplification from contamination was detected in the no template controls (NTCs).

### LAMP–BART droplets off-chip

LAMP–BART droplets with DNA template were analysed off-chip. Microfluidically-produced droplets were output into a petri dish in NOVEC 7500 oil containing 2 percent Picosurf surfactant overlaid with mineral oil.

**Figure 3:**
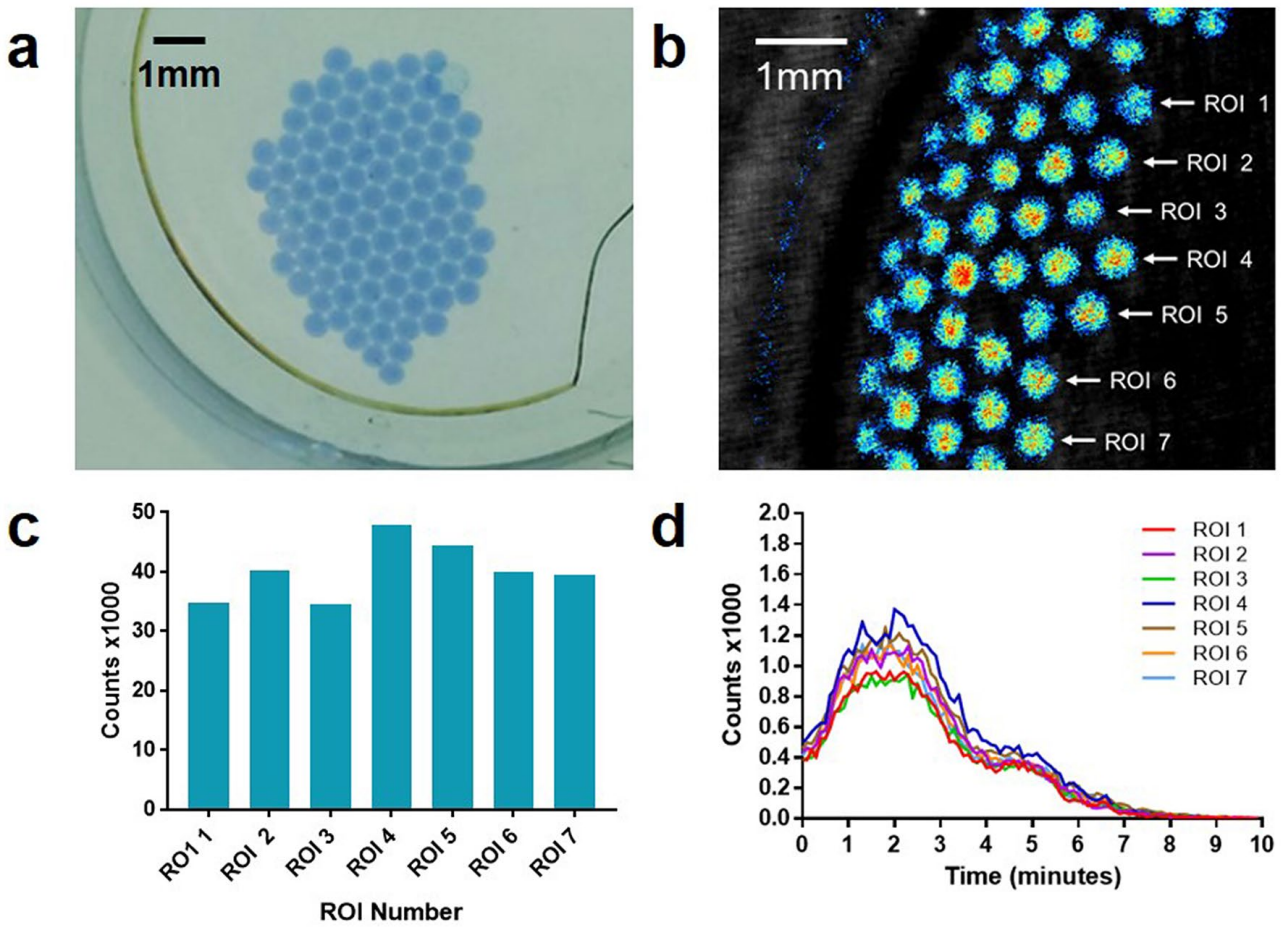
Multiple microfluidically produced surfactant stabilised LAMP–BART droplets are measured in a Petri dish. Artificial template (35Sp) at 2.6 × 10^8^ copies per droplet with 35Sp LAMP primers. Non-merging, discrete droplets were observed with a rapid bioluminescent light output evidencing successful DNA amplification (**a**) LAMP–BART droplets with blue dye (the dye was added for visualisation but removed from the experimental LAMP–BART reagent) in NOVEC PicoSurf under mineral oil photographed before transfer to photon imager, (**b**) pre-acquisition image showing the position of the droplets with total photon counts from the bioluminescent reactions overlaid. Regions of interest (ROIs) corresponding to individual droplets 1–7 are indicated, (**c**) total photon counts for each ROI, (**d**) real time photon counts from each ROI (Supplementary Information, thermostability; Fig. S3 and uniformity; Fig. S4, Video 3).

The LAMP–BART droplets clustered together without merging in the presence of Picosurf surfactant (Fig. 3a). Although care was taken to keep the droplets localised centrally in the well, the transfer to the imager caused movement to the side and this is evident from the change in droplet positions in the total photon count image (Fig. 3b). The bioluminescence from the droplets is discrete and regions of interest (ROIs) were assigned for seven nearest the centre. There is increased variability for the total photon counts for these ROIs (Fig. 3c) when compared to those droplets physically separated in tubing (Fig. 2). This variability is also evident with the real time photon counts which retain a rapid time-to-peak (average Tmax 1.65 ± 0.31 min) but without the smooth emission profile seen with physically constrained droplets, possibly due to the addition of surfactant affecting interfacial chemistry. The thermostability and uniformity of LAMP–BART droplets under mineral oil are shown in Supplementary Information Figs. S3 and S4 respectively.

### DNA gradient of LAMP–BART droplets

**Figure 4:**
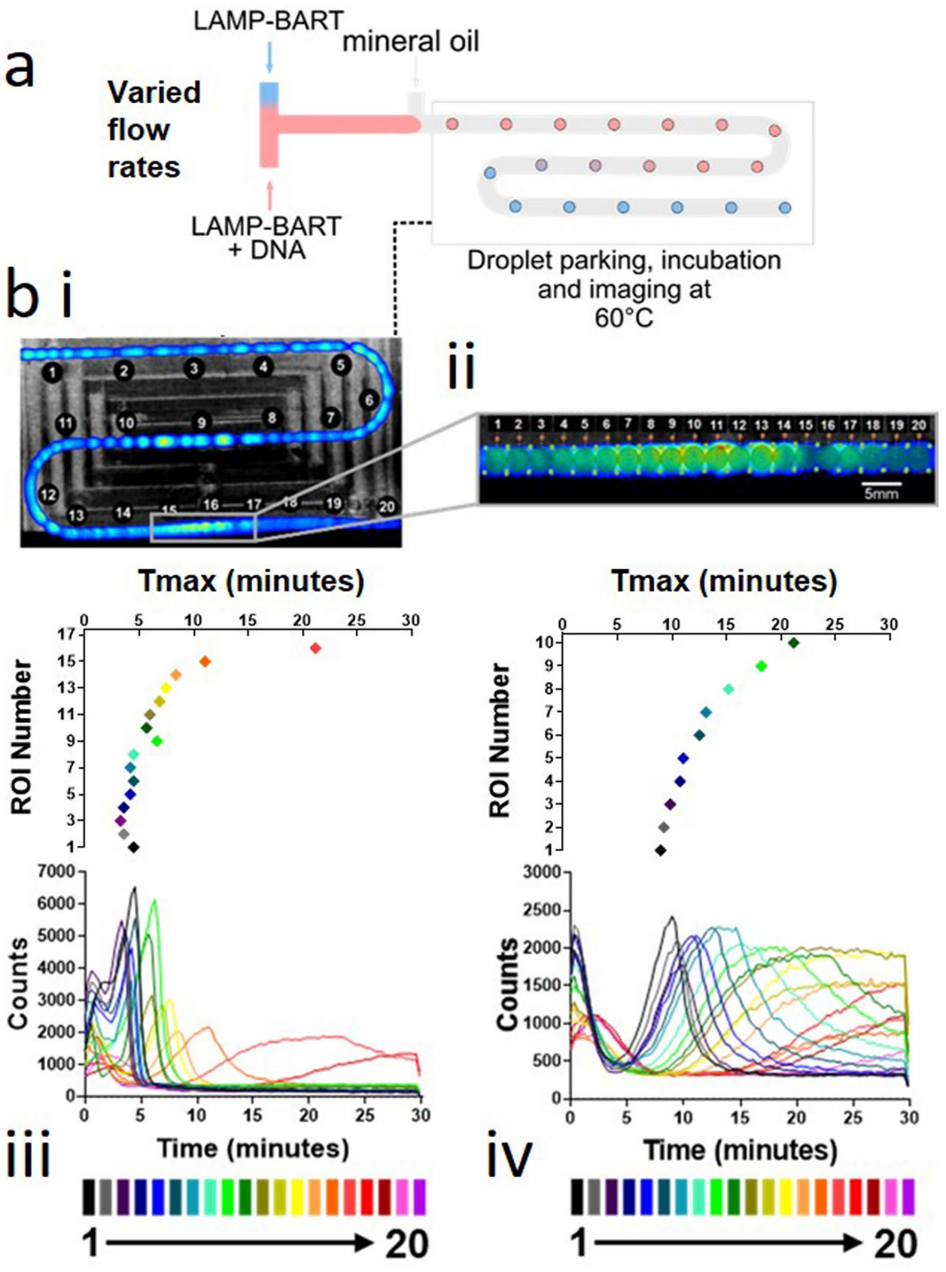
DNA template gradient within LAMP–BART droplets. Artificial template (35Sp) with input DNA concentration ranging from 8.7 × 10^9^ copies per droplet to 0 copies per droplet. Output droplets parked in tubing at 60 °C for 30 min. Time to peak light response decreases with increasing DNA concentration of the droplets. (**a**) schematic of the LAMP–BART droplet generation with controlled DNA template gradient achieved by gradient modulation control of the relative flow rates of two aqueous sample inputs at the first T-junction, ahead of droplet formation. (**b**i) Pre-acquisition image showing the position of the tubing in the temperature controlled device with total photon counts from the bioluminescent reactions overlaid, (**b**iii) regions of interests (ROIs) 1–20 indicating a wide range of DNA concentrations from approximately 8.7 × 10^9^ copies per droplet to the limit of detection and real time bioluminescence from each ROI, (**b**ii) subset of ROIs numbered 1–20 from area highlighted in (**b**i), (**b**iv) real time bioluminescence from this subset showing a narrower range of DNA concentrations. Amplification and detection of a range of DNA concentrations within the device by real time monitoring of light output is demonstrated with time to peak corresponding to initial DNA concentration. (Supplementary Information, Video 4, design of droplet water bath; Fig. S5, control experiment; Fig. S6).

LAMP–BART droplets with a gradient of DNA concentrations were generated. To demonstrate the control over the components forming the droplets and to show the DNA concentration dependent bioluminescent response, a DNA concentration gradient was established ranging from 8.7 × 10^9^ copies per droplet to 0 copies per droplet. A schematic of the droplet generation is shown in Fig. 4.

A DNA-template-positive LAMP–BART solution was mixed with a DNA-template-negative LAMP–BART solution at a varying ratio prior to droplet formation in order to generate LAMP–BART droplets of increasing concentration of DNA. These droplets were then parked in tubing for imaging. Selected ROIs from the full length of the tubing in the device were analysed to obtain BART peak data which showed initial rapid Tmax values (4.5 min) declining to slower and subsequently no observable peaks within the measurement timeframe (Fig. 4iii), as DNA template was diluted in successive droplets. The gradient was further interrogated between ROIs 14 and 17, where a further subset of ROIs between these markers was investigated highlighting the increase in Tmax with reducing concentration of DNA template (Fig. 4iv). The details of DNA gradient generation are in Supplementary Information Figs. S5 and S6.

### LAMP–BART in compartmentalised artificial cells

**Figure 5:**
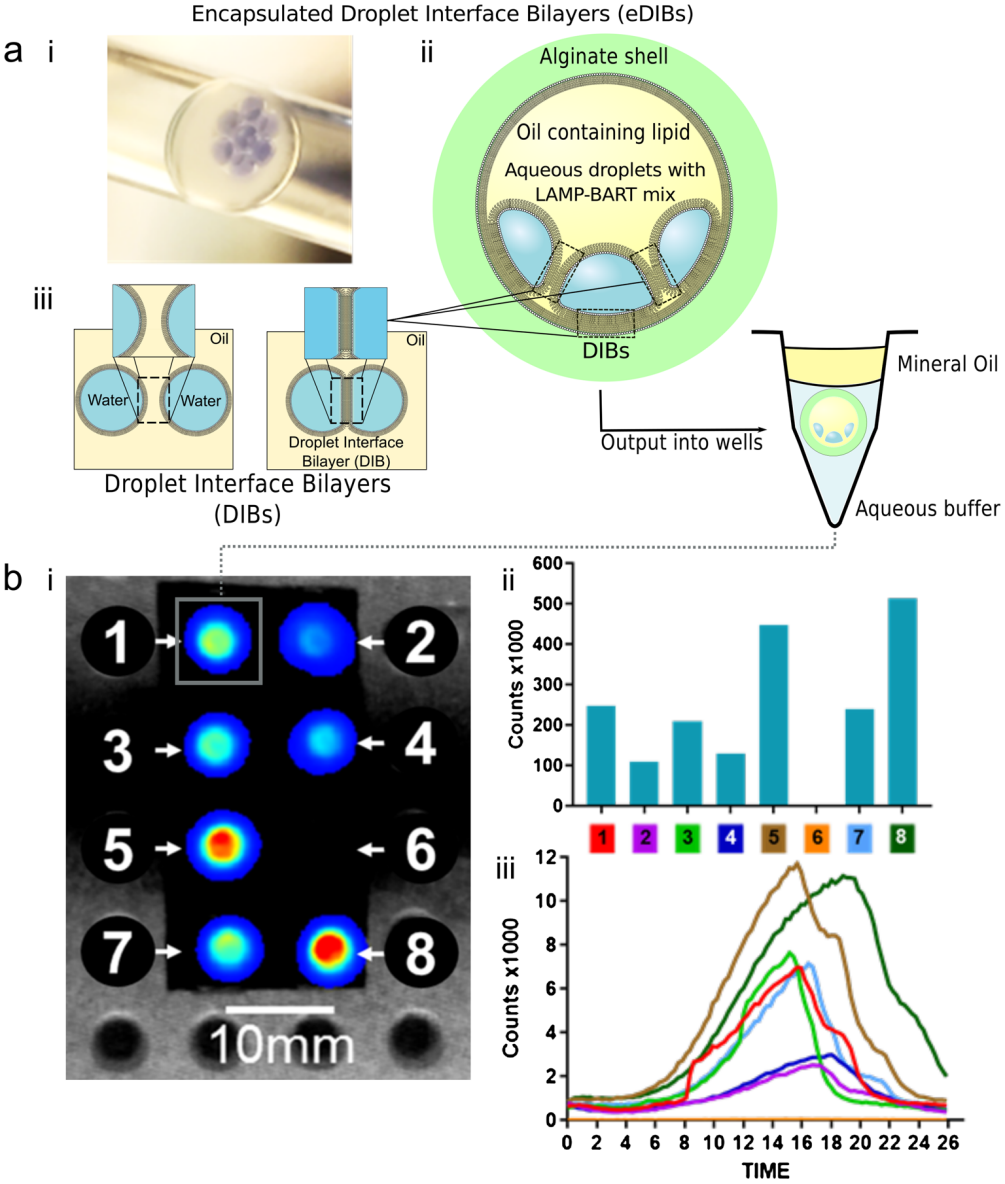
LAMP–BART encapsulated droplet interface bilayers (eDIBs). eDIBs are artificial cell mimics comprised of lipid membrane segregated droplet networks, encapsulated with a hydrogel shell. Artificial template (35Sp) at 2.6 × 10^8^ copies per droplet with 35Sp LAMP primers. (**a**i) photograph of an (eDIB) within microfluidic outlet tubing containing seven aqueous LAMP–BART droplet cores. (**a**ii,iii) Schematic of eDIB structure and principles of formation, self-assembled lipid monolayers at aqueous-oil interface are brought into contact to form artificial lipid bilayers, these are formed between aqueous droplets within an oil droplet, which is subsequently encapsulated in a hydrogel shell, where lipid bilayers also form where the internal droplets and hydrogel shell interfaces contact. The resultant structure comprises lipid bilayer segregated and stabilised droplet compartments within a hydrogel shell. These constructs were output from the microfluidic device into individual wells of a heating well plate (**b**i) image of total photon counts for eDIBs numbered 1–8, number 6 represents a control well where the eDIB was ruptured prior to imaging, (**b**ii) total photon counts from eDIBs 1–8 and (**b**iii) real time photon counts from each eDIB. The encapsulated LAMP–BART eDIB droplets are mechanically and thermally stable, and triggerable by the required reaction temperature. (Supplementary Information, Video 5; LAMP–BART eDIBs formed with DPhPC lipid only, Fig. S7).

LAMP–BART reagents and DNA template were encapsulated in droplets within a network of droplet interface bilayers. These droplets were microfluidically generated and encapsulated within a hydrogel shell (Fig. 5). Termed encapsulated droplet interface bilayers (eDIBs), they have been proposed as a chassis for artificial cells and freestanding analytical devices^47^. We showed the successful generation of eDIBs (Fig. 5) containing multiple droplets of LAMP–BART reagents and DNA template within the eDIB core. Furthermore, LAMP–BART droplets were stable within eDIBs, which were also tolerant of heating, allowing for DNA to be amplified and detected within such constructs. The microfluidically produced eDIBs were output into wells, heated to 60 °C and imaged, measuring the production of bioluminescent peaks characteristic of LAMP BART detection. This is in contrast to a control eDIB, which was deliberately ruptured within the well with a needle prior to heating, giving rise to coalescence between the internal droplets and the external buffer. The droplets within this eDIB coalesced with the surrounding aqueous environment within the well and no bioluminescence was recorded from this location (Fig. 5bi).

The eDIBs that were not ruptured remained stable at the LAMP–BART reaction temperature of 60 °C and throughout the 30 min assay. The location of the eDIBs is clear from the photon imaging (Fig. 5bi) however individual droplets within the eDIBs are not discernible using this imaging resolution. Baseline photon counting for each eDIB is broadly similar at the start of imaging acquisition. The variation in total photon counts (Fig. 5bii) from each eDIB is related to the BART peak heights at Tmax (Fig. 5biii) with counts at the BART peak ranging from 3000 to 12,000. All seven intact eDIBs showed that the LAMP–BART reaction was successful with an average Tmax of 16.64 ± 1.34 min. LAMP–BART eDIBs containing DPhPC lipid are shown in Supplementary Information Fig. S7.

### LAMP–BART in freestanding artificial cells with defined droplet numbers and tracking of bioluminescent signal

**Figure 6:**
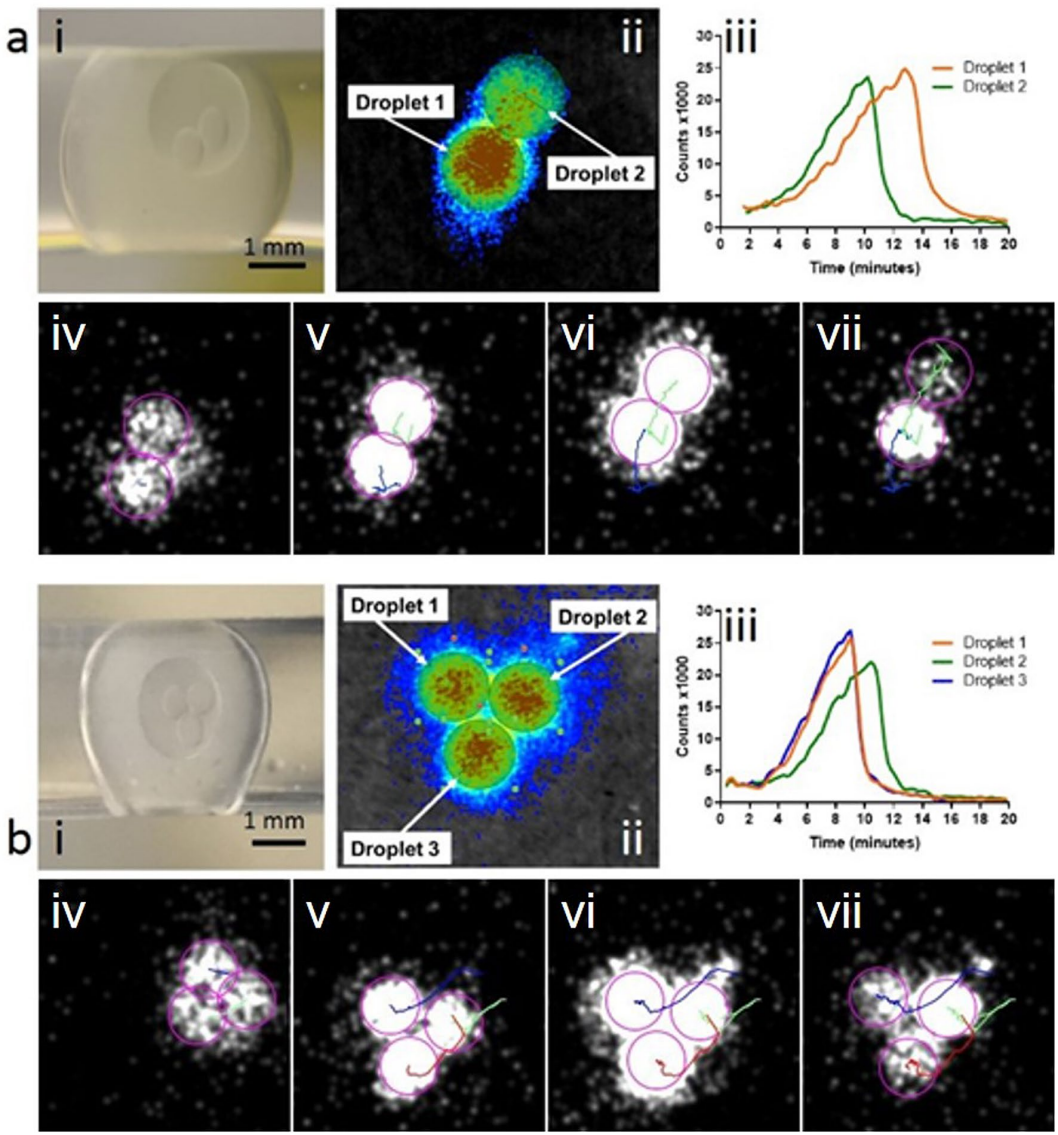
Two and three core LAMP–BART eDIBs with real-time tracking and quantification of light emission. Artificial template (35Sp) at 2.6 × 10^8^ copies per droplet with 35Sp LAMP primers. (**a**) Imaging of encapsulated droplet interface bilayer (eDIB) containing two internal droplets, (**b**) imaging of triple droplet eDIB, (i) pre-acquisition photographic images showing the eDIBs in the microfluidic tube following their production. These eDIBs were then output into aqueous containing wells where they remained unconstrained for the imaging of the LAMP–BART reaction. (ii) total photon counts images from the eDIBs with the positions of ROIs for real time analysis, (iii) real time bioluminescent output results from each Internal droplet of the eDIB extracted following tracking with ImageJ, (iv–vii) image sequence shows the successive movement and tracking of the eDIBs whilst DNA amplification and light emission takes place, with distinct signal measured from each membrane serrated aqueous droplet. Defined numbers of internal droplets can be generated and encapsulated in eDIBs with LAMP–BART eDIBs can be tracked in real time for accurate result from each cores. (Supplementary Information, Fig. S8; Video 6).

The ability to detect a unique LAMP–BART response for each droplet within an eDIB is demonstrated in Fig. 6. Here, eDIBs containing 2 and 3 internal aqueous cores are imaged using a macro imaging module on the Photon Imager, measuring a unique bioluminescent response for each of the droplets within the eDIBs. Here, individual internal droplets of eDIBs remain segregated by an adjoining lipid membrane. Lipid bilayers also separate the internal droplets from the encapsulating hydrogel shell, providing a bio-mimetic platform of membrane segregated compartments with the capability to harness membrane-mediated communication between neighbouring droplets and between droplets and the outside environment.

The total photon count images for the two and three core eDIBs show the movement of the droplets during the 20 min acquisition. The droplets were tracked using ImageJ software with the results off image intensity analysis following tracking are shown in Fig. 6, iii. The BART profiles are well defined with individual internal droplet tracking and quantification showing greater similarity to the BART profiles observed with droplets in tubing compared to the previous multiple core eDIBs measurements. The BART Tmax values for the two core eDIB were 12.8 and 10.2 min (average Tmax 11.5 ± 1.8) and 10.4, 9.0 and 9.0 min for triple core (average Tmax 9.5 ± 0.8). Results for single and quadruple core eDIBs are shown in Supplementary Information, Fig. S8 with raw images of the experiments, Fig. S9.

## Discussion

For the first time we have shown that microfluidic LAMP–BART droplets can be generated and that the light output from these droplets can be detected and quantified in real time. The uniformity of these droplets (Supplementary Information, Fig. S4) was evident from the consistent photon counts from each one and represents a proof-of-concept that nanoscale LAMP–BART volumes are possible. Furthermore, multiple droplets can be generated enabling quantification from large numbers of partitions, illustrating the potential to be developed into droplet digital, or low-copy number peak-variance LAMP quantification^16^ with dilute DNA samples. Positive LAMP–BART droplets were easily distinguishable in real time from negative control droplets, a feature important to a digital quantification approach.

Continuous observation of the dynamics of the bioluminescence provides rich information that informs on original DNA copy number in real time. This is a result of the combined effects of DNA amplification liberating PPi which subsequently generates ATP to energise bioluminescence, and at high concentrations PPi also serving to quench light emission. Thus the profile of light emission, rather than total photon counts, informs on the sequence copy number (Fig. 2bii; total photon counts, biii; maximum photon counts per droplet), yet retains a strong quantifiable signal regardless of initial template concentration. Thus the time to peak light emission informs on original copy number. By continuously imaging multiple droplets in parallel, large scalable measurements can be conducted. These multiple simultaneous measurements may be harnessed for assessment of multiple partitions of a single sample for digital or time variance methods to quantity low copy number, or at high copy number they may represent different samples or targets. Attractively, in principle one methodological approach can operate in either regime by simply creating a microfluidic dilution series.

The physical constraint of droplets in tubing coupled with the high time and pixel resolution of the Biospace PhotonIMAGER Optima produce high quality BART profiles (Fig. 2biii) which can be used to further study LAMP amplification and BART detection. The average time to peak for the eight droplets of 2.25 min is extremely rapid for a molecular diagnostic assay and consistent with a standard deviation of 3 seconds (0.05 min). Simple mineral oil was sufficient for the oil phase for in-channel measurement, but the propagation of parked droplets into a well required NOVEC 7500 oil with 2 percent PicoSurf surfactant to prevent droplet coalescence. At 52 °C evaporation could be controlled by a requisite layer of mineral oil. A greater number of droplets can be parked within a given area by this method but challenges still remain in preventing droplet fusion at higher reaction temperatures. The optimal temperature for LAMP–BART is 60 °C, determined by a compromise between Bst DNA polymerase activity and thermostability of the UltraGlo luciferase, and indeed higher temperatures for LAMP have been used with other detection methods. A broader peak was observed in these packed droplets off-chip (Fig. 3d) than when parked within a microfluidic channel, suggesting sub-optimal amplification possibly from the reduced assay temperature. Also the increased variation between Tmax values coupled with the broader BART peaks may be explained by differences at the droplet interface, for example from contact between droplets, air, surfactant, and plasticware.

The ability to control the components of the aqueous droplets as well as the volume, the rate of generation and the propagated number of droplets is important in such areas as DNA quantification, single copy number detection and synthetic biology. We showed in the generation of DNA gradients (Fig. 4) that the DNA composition of the droplets was controlled in conjunction with the flow rate and the droplet size. The increasing DNA concentration showed a decrease in Tmax values to less than 5 min for droplets at the high DNA concentration (approximately 10^9^ copies per droplet) end of the tubing. We note that the controlled dilution of a sample with LAMP–BART quantification could be used to remove the effect of an inhibitory substance such as SDS^11^ through dilution to gain a more accurate quantification.

The experiments performed here with eDIBs represent the first DNA amplification and detection reported in an artificial cell chassis, achieved here via LAMP coupled to bioluminescent reporting. LAMP–BART within eDIBs containing a number of internal cores (3-4 cores) displayed peaks in bioluminescence upon heating to 60 °C, confirming amplification of the DNA sample. The eDIBs were observed to remain intact during the course of the experiments, and droplet tracking imaging software could accommodate accurate quantification of light emission from individual cores even whilst unconstrained eDIBs moved during the course of the experiment.

Tmax for the eDIBs exhibited a larger variance (16.64 ± 1.34 min) in comparison to results obtained for aqueous droplets in oil (2.25 ± 0.05 min), as well as broader peaks. However, both droplets in eDIBs and aqueous droplets are of similar size and volume. This difference might be explained by heterogeneous heating in eDIBs, or disruptive interactions between the LAMP–BART reaction components and the lipid-rich droplet interface. Additionally, Tween-20 was removed from the LAMP–BART cocktail as it was expected that surfactant species would interfere with lipid bilayer formation at the droplet interfaces. It was also found that the incorporation of lipid with polyethylene glycol (PEG) attached to the lipid head, was an important factor in obtaining a cleaner LAMP–BART bioluminescence profile over time, as LAMP–BART occurring in eDIBs containing only the lipid DPhPC exhibited a more erratic bioluminescent profile as well as broader peaks (Supplementary Information, Fig. S7). Consequently a lipid cocktail composed of 50 percent DPhPC and 50 percent DSPE-PEG2000 was employed for the experiments reported here. This strategy was reported by Booth et al^43^. to optimise in vitro transcription and translation (IVTT) in lipid-bound droplets, and it is theorized that the PEG may provide a steric or electrostatic barrier between the droplet contents and the droplet interface, minimizing unwanted lipid-protein or lipid-nucleic acid interactions that might interfere with the LAMP–BART reaction.

A further experiment aimed at determining whether bioluminescence traces could be detected from individual eDIB cores, and for this eDIBs containing two or three cores were imaged using a macro lens (Fig. 6). The internal droplets exhibited unique bioluminescent profiles with an average Tmax of 16.28 ± 1.55 min for all 5 droplets measured, demonstrating that each droplet within an eDIB can be considered an individual assay unit. This may explain the broader peaks displayed in their collective measurement (Fig. 5), as they are likely to be an ensemble measurement of all of the droplets contained within.

These experiments provide a proof-of-concept for DNA to be amplified and detected within the droplets of an eDIB artificial cells. This represents a foundation for the use of eDIBs as stable, storable, self-contained analytical devices to detect specific DNA sequences in liquid samples. The multicompartment nature of the eDIB constructs may allow for multiple assays to take place within a single eDIB, for example. Or the network may be harnessed to combine with more complex chemical processing via the transport of molecular species through pores or channels within the lipid bilayers of the droplet network, linking otherwise segregated reaction chemistries. In this context, networked and sequential reactions in droplet networks have recently been demonstrated, using membrane proteins to control chemical communication and reaction initiation within such networks^49,50^. The ability to undertake sequence specific nucleic acid amplification and detection adds to the toolkit of available functional biochemistry in these systems. In combination, such approaches could be harnessed to transport DNA into internal compartments of eDIBs for subsequent LAMP amplification and BART detection. Similarly nanopore DNA sequencing approaches have been applied in droplet interface bilayers^51,52^ and could be combined with preceding in situ LAMP amplification within the droplet itself for efficient sample multiplexing^53^. In situ protein expression has been demonstrated in Droplet Interface Bilayer networks with subsequently expressed protein channels able to incorporate into the lipid bilayers^44–46^, in addition to demonstrations of in vitro gene circuits within droplet networks^54^. In situ LAMP–BART combined with these approaches could further enable complex multistep compartmentalised functionality more akin to biological cells. Furthermore, low copy DNA sequence specific triggers may be harnessed with LAMP within droplet networks, to generate and amplify coupled biochemical responses energised via the associated avalanched generation of pyrophosphate from the LAMP reaction which is subsequently converted to ATP. This is exemplified here in the form of light emission, with the temporal growth and subsequent inhibition of light emission determined by DNA concentration. In addition to application in soft-matter diagnostic devices, this approach could be harnessed as a generic reporting mechanism in smart artificial cells, or coupled to other ATP dependent biochemistries as a means for artificial cell energisation.

## Conclusions

Microfluidic LAMP–BART droplets provide stable, low volume, high throughput partitions for applications in DNA detection and quantification. Multiple droplets with uniformity of scale could form the basis of a digital LAMP–BART assay for absolute quantification of low concentrations of DNA targets from statistical analysis of positive and negative bioluminescent droplets. Smaller droplets would enable a greater number of individual reaction partitions from a sample volume for more accurate digital quantification. Increased sophistication of microfluidic liquid handling could ultimately enable analysis of multiple samples and multiple targets.

We achieved the incorporation of defined numbers of LAMP–BART droplets into eDIB artificial cells, enabling the reaction to be triggered by temperature and detected in real time by counting photons emitted. This control over the droplets and the development of more complex microfluidics would allow the incorporation of droplets composed of differing chemistries to provide the means to study, or exploit, complex DIB networking within individual eDIBs. The LAMP–BART eDIBs could be used as artificial cells to study their interaction with environmental conditions and provide the potential to transport DNA across the lipid bilayer to react with the LAMP–BART reagents. This would create the possibility of assessing multiple DNA targets in an array of eDIBs for the detection of single nucleotide polymorphisms (SNPs) and genetic variants in clinical samples.

## Methods

### LAMP–BART reagent preparation

Unless otherwise stated, all reagents were supplied by Sigma (Poole, United Kingdom). As previously described^14^ but with some modification, the LAMP amplification with BART detection reaction mixture contained 1X isothermal buffer (20 millimolar Tris-HCl, 10 millimolar ammonium sulphate, 50 millimolar potassium chloride (KCl), 2 millimolar magnesium sulphate, pH 8.8), 5 micrograms salmon sperm carrier DNA, 60 millimolar KCl, 0.4 milligrams per millilitre polyvinylpyrrolidone (PVP), 10 millimolar dithiothreitol (DTT), 300 micromolar each deoxynucleotide triphosphate (dNTP), 100 micrograms per millilitre D-luciferin (Europa, Ipswich, United Kingdom), 250 micromolar adenosine-5′-O-phosphosulfate (APS; Biolog, Bremen, Germany), 5.5 micrograms per millilitre Ultra-Glo luciferase (Promega, Madison, United States), 0.32 units per millilitre Bst polymerase v2.0 warm start (NEB), 0.375 units per millilitre ATP sulfurylase (NEB), 0.2 micromolar each displacement primer, 0.4 micromolar each Loop primer, 0.8 micromolar each LAMP primer and molecular grade water for a total reaction volume of 500 millilitres.

### Oligonucleotide primers and artificial templates

All oligonucleotide primers for LAMP amplification were synthesized and HPLC purified by Sigma (Supplementary Information, Table S1). All primers were diluted to a stock concentration of 100 micromolar and stored at − 20 °C. Artificial templates (Supplementary Information, Table S2) for 35S promoter and NOS terminator LAMP primers were synthesized by Integrated DNA Technologies (IDT; Iowa, United States) with PAGE purification. The 150 and 176 base oligonucleotides were diluted to (10 micromolar) and stored at − 20 °C. The artificial templates were designed to form looped secondary structures based on the cycling LAMP “dumbbells” to provide rapid amplification at high concentration. 35S promoter and NOS terminator LAMP assays were selected due to extensive previous studies with these primers^7,8,11,16^.

### Microfluidic reagent preparation

Mineral oil (Sigma) formed the oil phase for droplets in tubing (Fig. 2). For parked droplets, mineral oil was replaced with NOVEC 7500 2 percent w/w PicoSurf (Sphere Fluidics, Cambridge, UK) and the droplets were generated into NOVEC 7500 2 percent w/w PicoSurf under a layer of mineral oil in a cell culture plate (Eppendorf, Hamburg, Germany) (Fig. 3). For eDIBs the internal oil phase consisted of 4 milligrams per millilitre lipid solution (50 percent DPhPC and 50 percent DSPE-PEG2000; Avanti Polar Lipids, Alabama, USA) in hexadecane and silicone oil AR20 (2:1). The alginate phase consisted of 2 percent w/v low viscosity alginate with 50 milligrams per millilitre nanoparticulate calcium carbonate, adjusted to 0.1 molar ionic strength using sodium chloride. The external oil phase consisted of mineral oil with 0.5 percent v/v glacial acetic acid.

### Droplet propagation with infusion pumps

Two Graseby (Smiths Medical, Minneapolis, USA) 3150 automatic infusion pumps were modified to flow fluids from 10 millilitre Luer lock syringes connected with Luer to 10-32 coned connectors (Upchurch Scientific, IDEX, Illinois, USA) and fingertight fittings (IDEX) through 0.5 millimetre inner diameter (ID) fluorinated ethylene propylene (FEP) tubing (IDEX). Aqueous droplets were generated in the oil phase within a Tee junction (IDEX) with 1/16th of an inch outer diameter (OD) connectors. Lengths of tubing containing droplets were cut for analysis, or the droplets were flowed into a device. Lengths of tubing containing eight droplets were appropriate for the field of view with macro imaging.

### DNA gradient

In order to produce a gradient of DNA across a number of droplets, an additional T-junction was added upstream of the aqueous input into the droplet-generating T-junction. The inputs into this T-junction consisted of LAMP–BART solutions, one containing 1.3 × 10^11^ copies of the DNA target per microlitre, and the other containing no DNA. A LabVIEW (Austin, Texas, USA) 2017 protocol was used to control the syringe pump flow rates of both the LAMP–BART solutions to produce a continuous gradient of DNA concentration at a fixed total flow rate. The total output flow rate was maintained at 1 millilitre per hour in order to ensure consistent droplet generation at the downstream T-junction where the oil phase was mineral oil at 2 millilitre per hour.

### Production of eDIBs

The eDIBs were produced using a purpose-built microfluidic device as described^47^. Fluids were flowed using Legato series syringe pumps (KD Scientific, USA), at a rate of 1:8:150:200 millilitre per hour, for the internal aqueous, lipid in oil, alginate, and acid in oil solutions, respectively. This gave rise to eDIBs containing 1 or 2 internal aqueous cores, and by changing the internal aqueous flow rate to 2 millilitre per hour, eDIBs were produced with 3–4 cores. The eDIBs were then parked in the final FEP tube of the microfluidic device and outputted individually into wells by flowing the alginate fluid at a manageable flow of 50 millilitre per hour.

### DNA amplification

The warm start displacement polymerase is activated above 45 °C triggering the LAMP amplification using a Torrey Pines (San Diego, California, USA) modified programmable heat block set at 52 or 60 °C. The cut lengths of tubing and cell culture plates with parked droplets were placed directly onto the heated surface. Pre-heated glycerol at 60 °C was added to the water bath device on the heat block to start the LAMP reaction. The cut tubing, parked droplets and eDIBs were carefully disposed of post amplification to reduce contamination risk.

### Bioluminescent imaging

All imaging of the bioluminescent LAMP–BART reaction in real-time utilised the PhotonIMAGER Optima (Biospace Lab, Nesles-la-Vallée, France). Either the 55 millimetre lens or macro module, for increased pixel resolution, was used depending on the required field of view. Pre-acquisition illuminated imaging was used to locate the droplets and eDIBs and provide a background for the bioluminescent acquisition images. Imaging was terminated manually after BART peaks had been observed, between 10 and 30 min from the start. For large data files, BVR reduction software (Biospace Lab, Nesles-la-Vallée, France) was used to reduce the number of pixels used for analysis. The data was analysed using M3 Vision software (Biospace Lab, Nesles-la-Vallée, France) version 1.1.2.26170, Microsoft Excel and Graphpad PRISM version 8.3.

### Real-time tracking of bioluminescence

The quantification of LAMP–BART in individual cores of eDIBs was performed by conducting individual core-tracking using the ImageJ plugin trackmate^55^ included in the FIJI distribution of ImageJ (version 2.0.0)^56^. Spatially correlated photon counting data from the Photon Imager was exported as TIFF image stack, with data integrated over a period of 1200 seconds to give a frame rate of 1 frame per 13.3 seconds. To enhance tracking efficiency, signal to noise was enhanced by employing a Gaussian blur (width = 2 pixels, depth = 1 frame) to minimize background noise prior to tracking. Trajectory linking employed maximum linking and gap-closing distances of 20 pixels, with a maximum gap-closing frame gap of two frames. Intensity data was then extracted from the tracked bioluminescent cores for quantitative analysis.

## Supplementary information

Supplementary Information.

Supplementary Video 1.

Supplementary Video 2.

Supplementary Video 3.

Supplementary Video 4.

Supplementary Video 5.

Supplementary Video 6.

## Data Availability

The datasets generated during and/or analysed during the current study are available from the corresponding author on reasonable request.
