# Author Correction: Bioluminescent detection of isothermal DNA amplification in microfluidic generated droplets and artificial cells

**DOI:** 10.1038/s41598-021-90099-5

**Published:** 2021-05-18

**Authors:** Patrick Hardinge, Divesh K. Baxani, Thomas McCloy, James A. H. Murray, Oliver K. Castell

**Affiliations:** 1 Cardiff School of Biosciences, Cardiff University, Sir Martin Evans Building, Museum Avenue, Cardiff, CF10 3AX UK; 2 Cardiff School of Pharmacy and Pharmaceutical Sciences, Cardiff University, Redwood Building, King Edward VII Avenue, Cardiff, CF10 3NB UK; 3 Cardiff School of Engineering, Queen’s Buildings, 12‑17 The Parade, Cardiff, CF24 3AA UK; 4 Molecular Sciences Research Hub, Imperial College London, 80 Wood Lane, Shepherd’s Bush, London, W12 0BZ UK

Correction to: *Scientific Reports* 10.1038/s41598-020-78996-7, published online 14 December 2020

The original version of this Article previously published contained lower resolution images for Figures 1 and 5.

In addition, the last paragraph of the Discussion section was a duplication of information contained in the second last paragraph and has now been removed.

The original last paragraph is provided below.

“These experiments provide a proof-of-concept for DNA to be amplified and detected within the droplets of an eDIB artificial cells. This represents a foundation for the use of eDIBs as stable, storable, self-contained analytical devices to detect specific DNA sequences in liquid samples. Membrane protein channels have been shown to be able to insert in the lipid bilayers of eDIBs^47^, and could be used to transport DNA into its internal compartments for subsequent LAMP amplification and BART detection. Similarly nanopore DNA sequencing approaches have been applied in droplet interface bilayers^51,52^ and could be combined with preceding in situ LAMP amplification within the droplet itself. DNA expression through in vitro transcription and translation (IVTT) has been demonstrated in similar droplet interface bilayers systems, along with the ability to incorporate subsequently expressed protein channels in their lipid bilayers^44,45,46^, in combination this would enable multistep compartmentalised functionality akin to biological cells. In this regard, the multicompartment nature of the eDIB constructs may allow for multiple assays to take place within a single eDIB, for example. Or for more complex chemical processing to occur via the transport of molecular species through pores or channels within the lipid bilayers of the droplet network to link otherwise segregated reaction chemistries. Furthermore, low copy DNA sequence specific triggers may be harnessed with LAMP, to generate and amplify coupled biochemical responses energised via the associated avalanched generation of pyrophosphate from the LAMP reaction which is subsequently converted to ATP. This is exemplified here in the form of light emitting artificial cells, with the temporal growth and subsequent inhibition of light emission determined by DNA concentration. In addition to application in soft-matter diagnostic devices for DNA detection, this approach could be harnessed as a generic reporting mechanism in smart artificial cells, or coupled to other ATP dependent biochemistries as a means for artificial cell energisation.”

The original Article has been corrected.

