# Supplementary material for: Bioluminescent detection of isothermal DNA amplification in microfluidic generated droplets and artificial cells
Source: Sci Rep. 2020 Dec 14;10:21886. doi: 10.1038/s41598-020-78996-7 (PMC7736893; doi:10.1038/s41598-020-78996-7)
Supplement: Supplementary file 1 — Supplementary Information. [file 41598_2020_78996_MOESM1_ESM.pdf]

Supporting Information

Bioluminescent Detection of Isothermal DNA Amplification in Microfluidic Generated Droplets and Artificial Cells

Patrick Hardinge[1,*], Divesh K. Baxani[2,4], Thomas McCloy[2,3], James A. H. Murray[1] and Oliver K. Castell[2]

[1]Cardiff School of Biosciences, Sir Martin Evans Building, Cardiff University, Museum Avenue, Cardiff, CF10 3AX, United Kingdom
[2]Cardiff School of Pharmacy and Pharmaceutical Sciences, Redwood Building, Cardiff University, King Edward VII Avenue, Cardiff, CF10 3NB, United Kingdom
[3]Cardiff School of Engineering, Queen's Buildings, 14-17 The Parade, Cardiff CF24 3AA, United Kingdom
[4]Molecular Sciences Research Hub, Imperial College London, 80 Wood Lane, Shepherd's Bush, London, W12 0BZ, United Kingdom
*corresponding author: hardingep@cardiff.ac.uk

Table of Contents

1. LOD of LAMP-BART assay of NOSt artificial DNA template in droplets
2. LAMP-BART droplet thermostability
3. LAMP-BART droplet uniformity
4. DNA gradient generation
5. LAMP-BART eDIBs containing DPhPC lipid only
6. One and four core eDIBs
7. Raw images
8. Video files
9. Tables

1. LOD of LAMP-BART assay of NOSt artificial DNA template in droplets

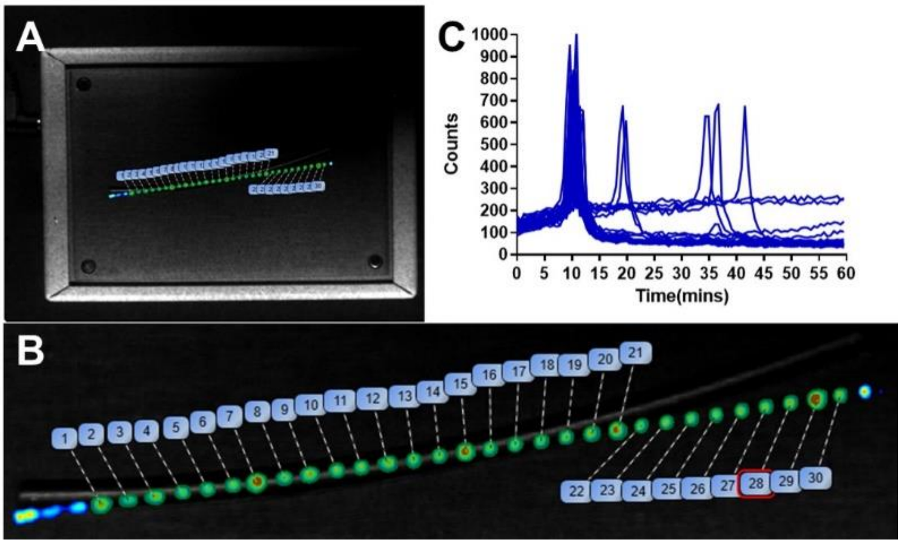

Figure S1: Multiple LAMP-BART droplets to determine the limit of detection (LOD) of NOSt LAMP assay with NOSt artificial DNA template. Concentration of artificial template in droplets 10fM. **A:** Position of LAMP-BART droplets within tubing on heating block. **B:** Zoom of selected ROIs (n=30). **C:** Real-time photon counts from each ROI. 93% amplification frequency with average Tmax 14.1 minutes and standard deviation 8.7 minutes. Limit of detection is calculated to be approximately 4 copies per droplet.

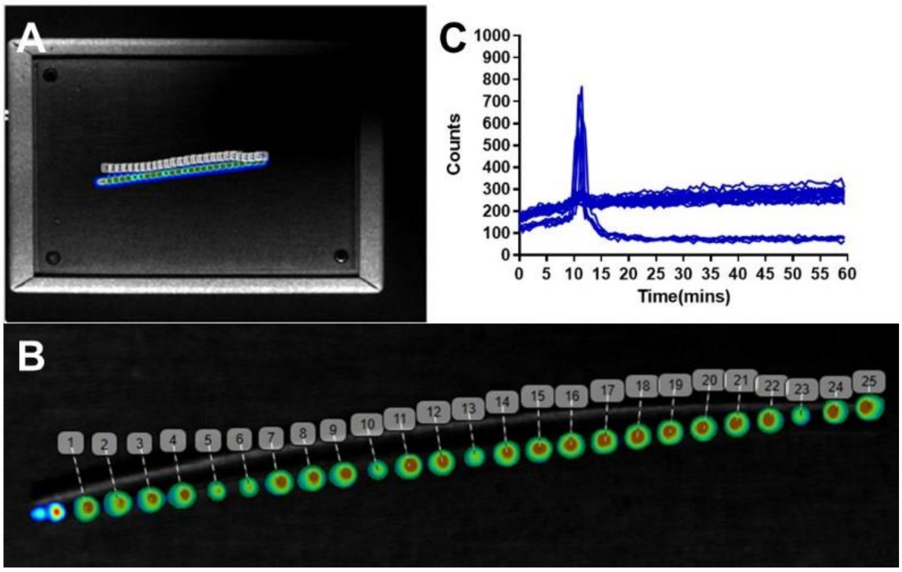

Figure S2: Multiple LAMP-BART droplets to determine the limit of detection (LOD) of NOSt LAMP assay with NOSt artificial DNA template. Concentration of artificial template in droplets 1fM. **A:** Position of LAMP-BART droplets within tubing on heating block. **B:** Zoom of selected ROIs (n=25). **C:** Real-time photon counts from each ROI. 20% amplification frequency with average Tmax 11.0 minutes and standard deviation 0.3 minutes. Calculation of copy number ~0.4 copies per partition.

2. LAMP BART droplet thermostability

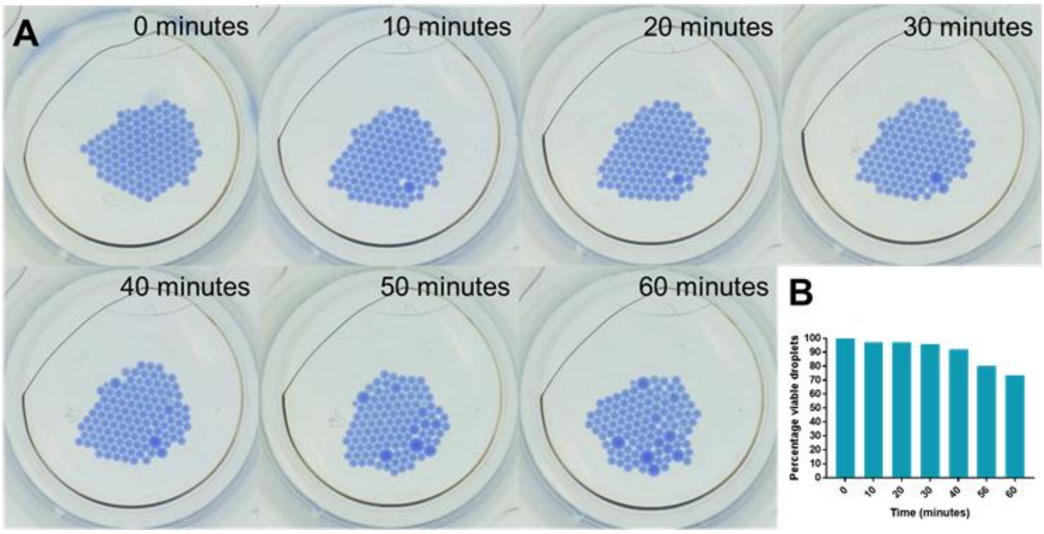

Figure S3: LAMP-BART droplet thermostability. Parked LAMP-BART droplets generated in NOVEC PicoSurf propagated into NOVEC PicoSurf under mineral oil. The isothermal buffer in the LAMP BART reagent contained 1 percent Span80. Assay temperature 52 degrees C for 60 minutes (the blue dye was added for visualisation of the droplets). **A:** LAMP-BART droplets at time points 0 to 60 minutes. **B:** Percentage viable droplets at each time point.

3. LAMP-BART droplet uniformity

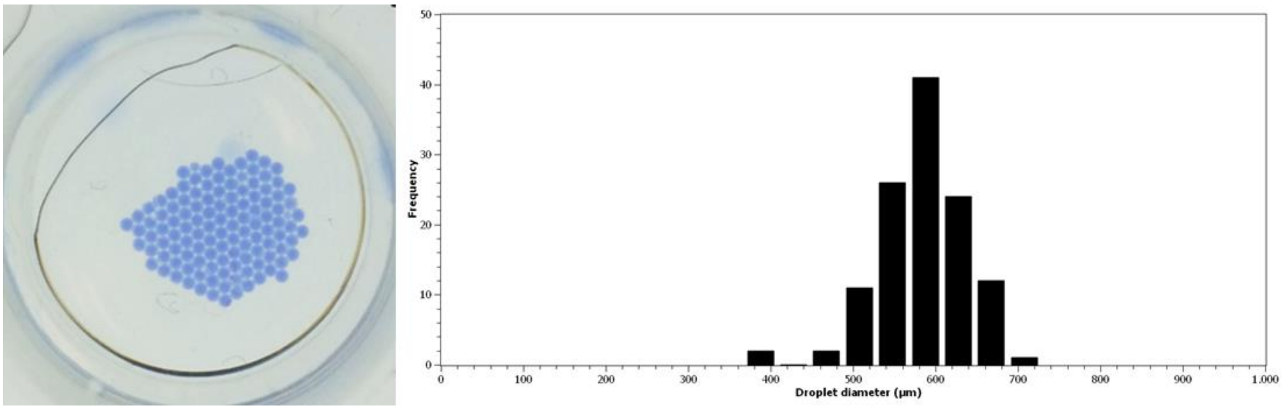

Figure S4: Droplet uniformity. Size (diameter) uniformity of outputted LAMP-BART droplets expressed as a frequency distribution (n = 119).

4. DNA gradient generation

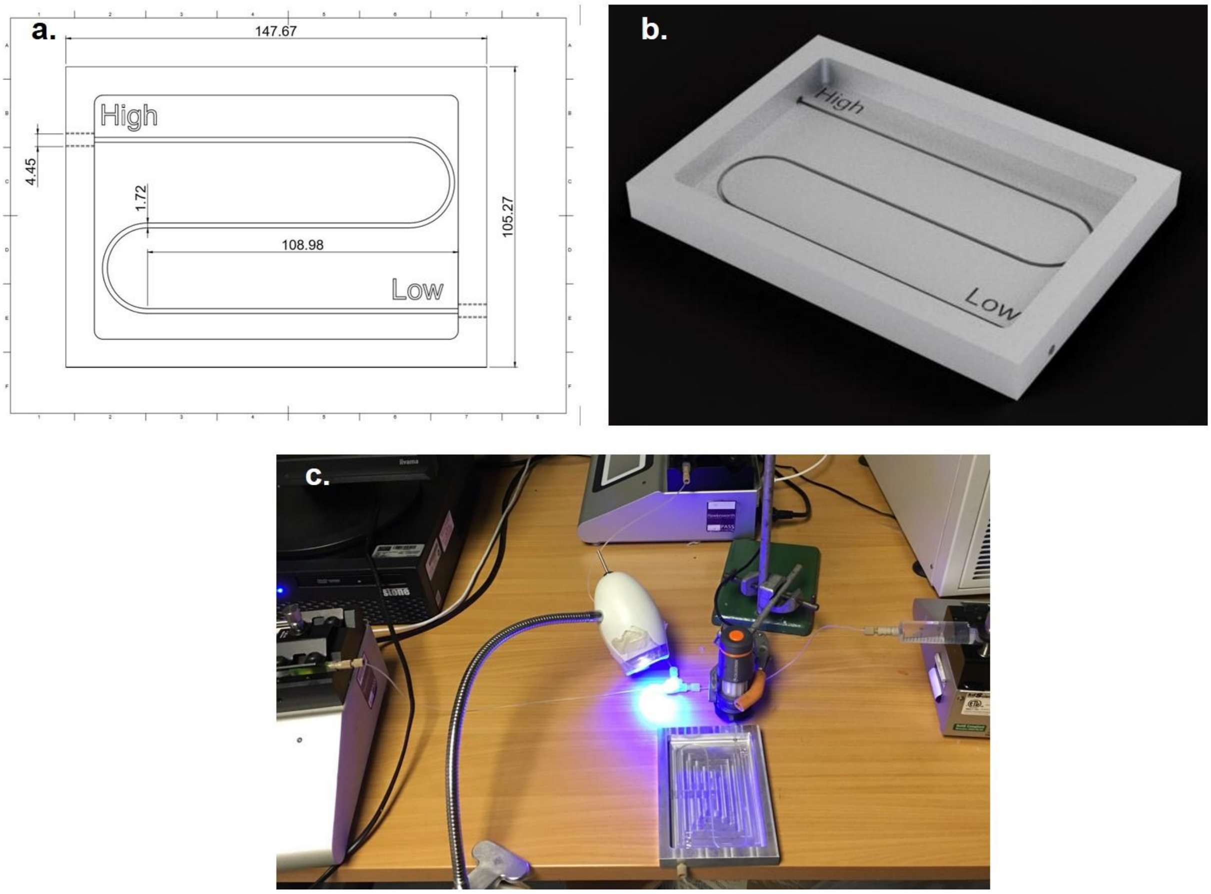

Figure S5. **A:** Dimensions (millimetres) of the droplet water bath used to create the DNA gradient. **B:** Render of the water bath device showing the space available for the pre-heated glycerol to be added for DNA amplification. **C:** Set-up of the control experiment used to validate the creation of the gradient.

A DNA gradient across droplets was generated by varying the flow rates of a LAMP-BART solution with and without DNA template. This was achieved by implementing a LabVIEW 2017 program (Figure S3a). LAMP-BART without DNA was linearly ramped down whilst LAMP-BART with DNA (target concentration = 1.3 x 10^{14} copies per millilitre) was linearly ramped up, resulting in an increasing concentration of DNA over time. The total flow rate was maintained at 1 millilitres per hour to ensure consistent droplet generation. The ability to form a concentration gradient over time in this manner was validated by using deionized water and a 0.1 millimolar calcein solution as the aqueous inputs, and ramping down the former and ramping up the latter, resulting in an increasing concentration of calcein across a series of droplets. This was measured by extracting the green pixel intensity value of the droplets generated using ImageJ (Figure S3b).

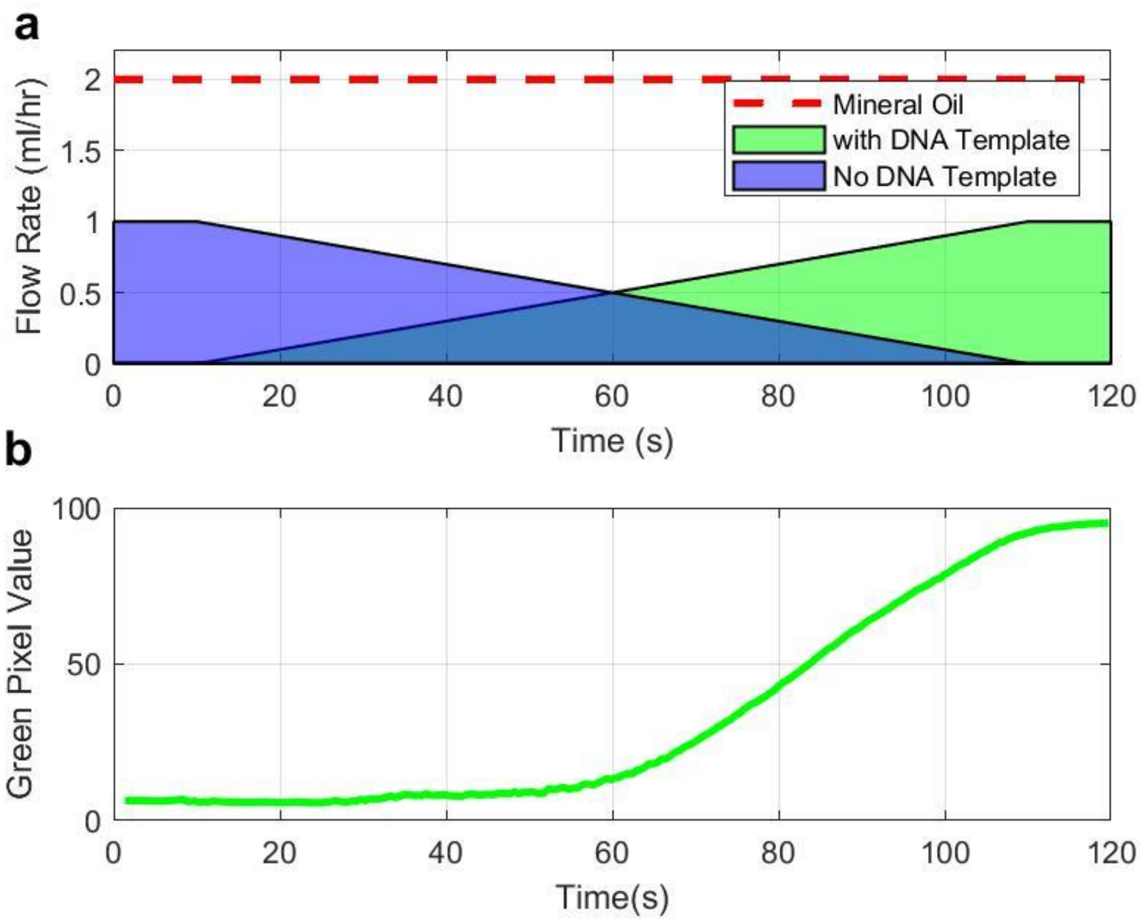

Figure S6. **A:** Programmed flow rates for the generation of the DNA gradient. **B:** 0.1 millimolar of Calcein in DI water was used to validate the generation of a gradient across droplets. The measured green colour level during the formation of the gradient under illumination from a blue LED lamp corresponds to the concentration of calcein across numerous droplets. The delay in onset to increased fluorescence in B corresponds to the time delay for fluid flow from the two-stream mixing geometry to the image capture area following droplet formation.

5. LAMP-BART experiments in eDIBs containing DPhPC lipid only

Prior to the use of DSPC-PEG2000 lipid in eDIBs, LAMP-BART was performed in eDIBs using DPhPC lipid only. The bioluminescent profile indicated that the LAMP-BART reaction was occurring, although the peaks were significantly broader than those in simple droplets (Figure 4). It was suspected that this may have been caused by unfavourable electrostatic or steric interactions between protein components of the LAMP-BART reaction and the lipid monolayer and bilayer at the droplet interface. This is similar to observations reported by Booth et al[1] for an *in vitro* transcription and translation system in DIBs.

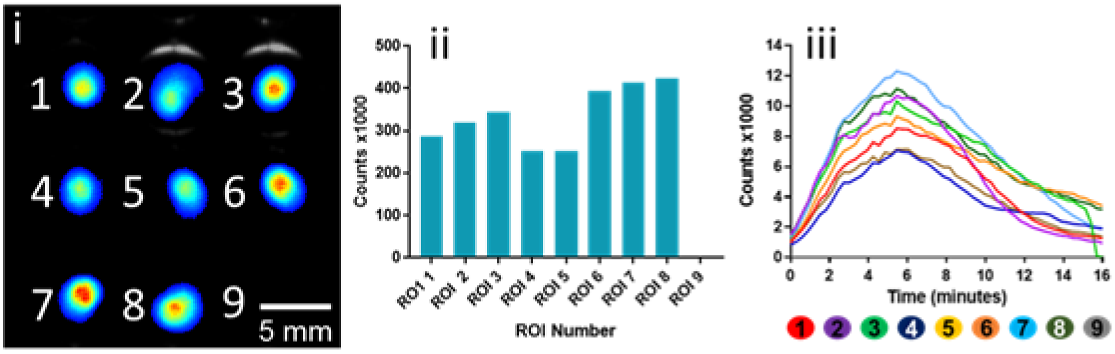

Figure S7: LAMP-BART encapsulated droplet interface bilayers (eDIBs) using DPhPC lipid. **i:** image of total photon counts for eDIBs numbered 1 to 9, number 9 was ruptured before imaging, **ii:** total photon counts from eDIBs 1 to 9 and **iii:** real time photon counts from each eDIB.

6. One and four core eDIBs

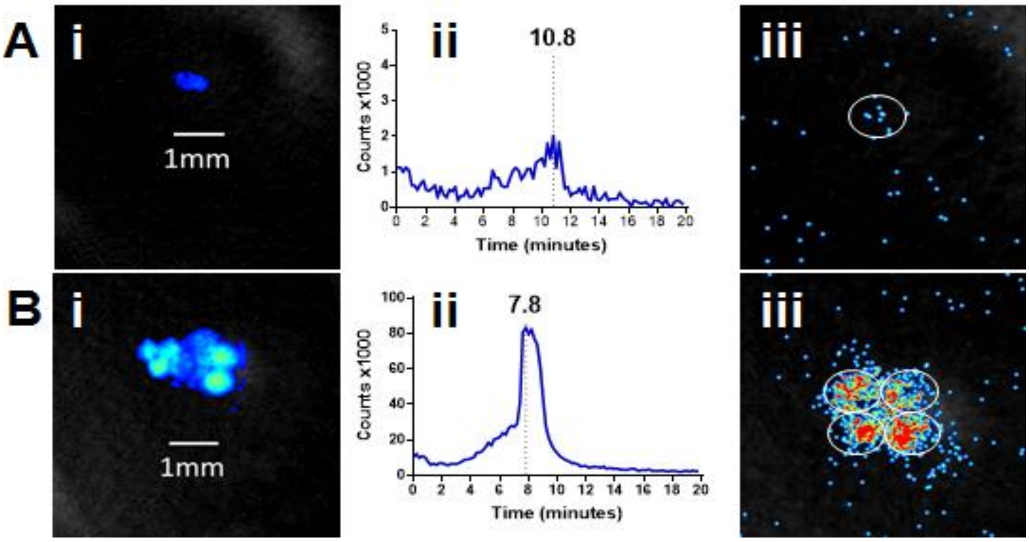

Figure S8: One and four core eDIBs. **A:** eDIB containing a single LAMP-BART droplet, **B:** four droplet LAMP-BART eDIB. **i:** image of total photon counts **ii:** real time photon counts **iii:** single time point to show single or quadruple cores.

7. Raw Images

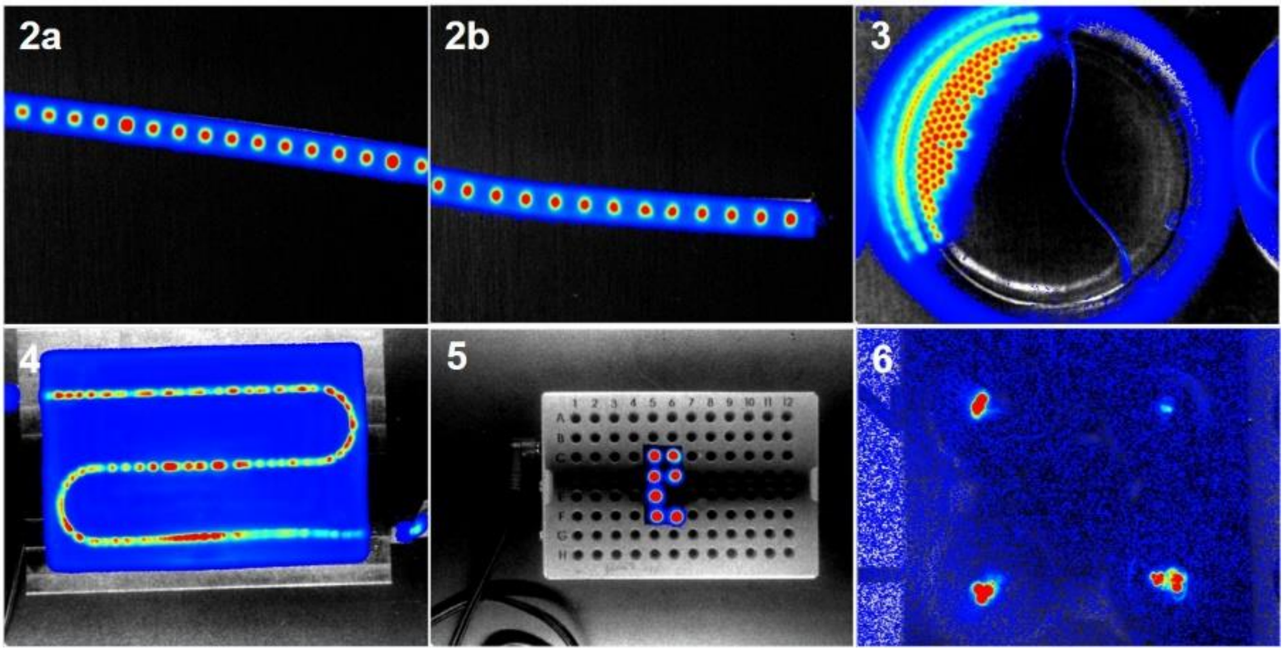

Figure S9: Raw total photon count images from the PhotonIMAGER Optima viewed with M3 Vision software (Biospace Lab, Nesles-la-Vallée, France). Each photon is time stamped enabling real time analysis of the data. Figure 2: LAMP-BART droplets in the absence (2a) and presence (2b) of DNA template. Figure 3: Multiple microfluidically produced surfactant stabilised LAMP-BART droplets (3) are measured in a petri dish. Figure 4: DNA template gradient within LAMP-BART droplets (4). Figure 5: LAMP-BART (5) encapsulated droplet interface bilayers (eDIBs). Figure 6: Two and three core LAMP-BART eDIBs (6) with real-time tracking and quantification of light emission.

8. Video files

Video 1: LAMP-BART droplets in tubing - no DNA template control (Figure 2a)
(assay duration 600s, frames 1 per 10s, 60 frames, final video 2 frames per second)
Video 2: LAMP-BART droplets in tubing - positive DNA template control (Figure 2b)
(assay duration 1800s, frames 1 per 10s, 180 frames, final video 10 frames per second)
Video 3: LAMP-BART parked droplets (Figure 3)
(assay duration 600s, frames 1 per 10s, 60 frames, final video 3.3 frames per second)
Video 4: LAMP-BART DNA gradient in tubing (Figure 4)
(assay duration 1800s, frames 1 per 10s, 180 frames, final video 10 frames per second)
Video 5: LAMP-BART eDIBs (Figure 5)
(assay duration 1570s, frames 1 per 10s, 157 frames, final video 5.2 frames per second)
Video 6: One to four core LAMP-BART eDIBs (Figure 6)
(assay duration 1200s, frames 1 per 10s, 120 frames, final video 5 frames per second)

9. Tables

Target	Primer type	Notation	Primer sequence (5' to 3')
35Sp	Displacement	F3	CTTATATAAGAGGAAGGGTCT
35Sp	Displacement	B3	GATAAAGGAAAGGCTATCATT
35Sp	LAMP	FIP	CCACGTCTTCAAAGCAAGTGG-TTTT-GGATAGTGGGATTGTGCGTC
35Sp	LAMP	BIP	TTCCACGATGCTCCTCG-TTTT-CCTCTGCCGACAGTGG
35Sp	Loop	LoopF	TCCACTGACGTAAGGG
35Sp	Loop	LoopB	GGGGTCCATCTTTGGG
NOSt	Displacement	F3	CGCGATAATTTATCCTAGTTTG
NOSt	Displacement	B3	CGTTCAAACATTTGGCAAT
NOSt	LAMP	FIP	GCATGACGTTATTTATGAGATTTTTCGCGCTATATTTTGTTTTCTA
NOSt	LAMP	BIP	CATGCTTAACGTAATTCAACATTTTTGAATCCTGTTGCCGGTC
NOSt	Loop	LoopF	GATTAGAGTCCCGCAATTATAC
NOSt	Loop	LoopB	AAATTATATGATAATCATCGCAA

Table S1. LAMP primers for 35S promoter and NOS terminator sequences. The primer sequences have been previously described and are attributed to Lee 2009[2] and Kiddle 2012[3].

Target sequence	Template sequence (5' to 3')
35Sp BIP	CCACGTCTTCAAAGCAAGTGGGGATAGTGGGATTGTGCGTCCCCTTACGTCAGTGG ACCACTTGCTTTGAAGACGTGGTCTAGATTCCACGATGCTCCTCGGGGGTCCATCTT TGGGCCACTGTCGGCAGAGGCGAGGAGCATCGTGGAA
NOSt FIP	CATGCTTAACGTTAATTCAACATGAATCCTGTTGCCGGTCTTGCGATGATTATCATAT AATTTTGTTGAATTAACGTTAAGCATGTCTAGAGCATGACGTTATTTATGAGATGATT AGAGTCCCGCAATTATACTAGAAAACAAAATATAGCGCGATCTCATAAATAACGTCAT GC

Table S2. Artificial DNA template sequences for 35S promoter and NOS terminator LAMP primers.

References

1 M.J. Booth, V.R. Schild, A.D. Graham, S.N Olof and H. Bailey, Science Advances, 2016, **2**, e1600056.

2 D. Lee, M. La Mura, T. R. Allnutt and W. Powell, *BMC Biotechnology, 2009,* **9**:7.

3 G. Kiddle, P. Hardinge, N. Buttigieg, O. Gandelman, C. Pereira, C. J. McElgunn, M. Rizzoli, R. Jackson, N. Appleton, C. Moore, L. C. Tisi and J. A. Murray, *BMC Biotechnol*ogy, 2012, **12**:15.
